# 6q24 Transient Neonatal Diabetes – How to Manage while Waiting for Genetic Results

**DOI:** 10.3389/fped.2016.00124

**Published:** 2016-11-17

**Authors:** Julie Fudvoye, Khaldoun Farhat, Virginie De Halleux, Corina Ramona Nicolescu

**Affiliations:** ^1^Department of Paediatric, University of Liège, Liège, Belgium; ^2^Medical School of University of Liège, Liège, Belgium; ^3^Department of Neonatology, Centre Hospitalier Regional de la Citadelle, Centre Hospitalier Universitaire de Liège, Liège, Belgium; ^4^Department of Pediatric Endocrinology, Centre Hospitalier Regional de la Citadelle, Liège, Belgium

**Keywords:** intrauterine growth restriction, transient neonatal diabetes, chromosome 6q24

## Abstract

Diabetes, rare in the neonatal period, should be evoked in every newborn presenting with unexplained intrauterine and early postnatal growth retardation. This case report illustrates the clinical course and therapeutic approach of a newborn diagnosed with transient diabetes. The baby was born at 37 weeks of gestation with a severe intrauterine growth restriction. Except a mild macroglossia and signs of growth restriction, physical examination was normal. On the fifth day of life, hyperglycemia (180 mg/dl) was noted, and the next day, the diagnosis of diabetes was confirmed (high blood sugar, glucosuria, undetectable levels of insulin and C-peptide). Insulin infusion, initially intravenously and then subcutaneously, was started, tailored to assure the growth catch-up and normalize the blood sugar levels. At the age of 4 weeks, the baby returned at home under pump. At 8 weeks, the clinical impression of evolution to a transient diabetes (decreasing needs of insulin with very satisfactory weight gain) was genetically confirmed (paternal uniparental disomy of chromosome 6). There is no screening for neonatal diabetes, but the clinical suspicion avoids the metabolic decompensation and allows early initiation of insulin therapy. The genetic approach (for disease itself and its associated features) relies on timely clinical updates.

## Introduction

Neonatal diabetes is a differential diagnosis to be evoked in newborns at term with intrauterine growth restriction (IUGR) and early unsatisfactory postnatal weight evolution. Its genetic characterization can be available in 1–2 weeks and guides the therapeutic approach. Two subgroups are defined today on clinical and genetic background: transient and permanent neonatal diabetes mellitus.

Approximately 50% of the neonatal diabetes cases require lifelong treatment to control glycemia (permanent diabetes), and for the remaining half of the cases, a spontaneous remission is observed, usually within the first 3 months (transient diabetes), and a clinical relapse is always possible (1).

Early diagnosis based on the clinical and biological background avoids the metabolic catastrophe of ketoacidosis and allows early insulin therapy initiation. There are no guidelines for diabetes management during the first months of life, but two remarks are of unquestionable value – these babies need insulin with adequate or high caloric intake to assure a satisfactory weigh gain and, in the era of advanced technology, insulin administration could partially mimic the pancreas physiology. The availability of genetic results (in a couple of weeks) definitively changed the short- and long-term management of these babies.

We present a case of transient neonatal diabetes (complete loss of methylation on chromosome 6q24) managed mostly by continuous subcutaneous insulin infusion (CSII), with very short hospital stay (3 weeks). The immediate and at distance evolution was favorable, with complete restoration of anthropometric parameters at the age of 3 months and no hyper- or hypoglycemic events.

## Case Report

A 5-day-old newborn boy was transferred to our Neonatal Intensive Care Unit for unfavorable postnatal growth progression, in absence of any symptoms. He was born at 37 weeks of gestation and presented a severe IUGR with birth weight 1980 g (<3rd percentile), length 43 cm (<3rd percentile), and head circumference 30 cm (<3rd percentile on WHO chart). His both parents are healthy, and the overall medical family history is irrelevant. On physical examination, he was well-appearing, with normal vital signs and normal hydration status, but the subcutaneous tissue was underdeveloped. There were no dysmorphic features, unless a mild macroglossia. Neurologically, the baby was relatively well, but a certain hypotonia was noted. He was breastfed with normal suction reflex but poor weight gain.

During the first days of life, routine blood sugar monitoring performed for newborn with IUGR was normal. Laboratory investigations did not find any explanation for the severe IUGR.

On the fifth day, the capillary blood sugar was at 180 mg/dl, with no concomitant glucosuria, in the absence of any stress. The venous blood sugar collected the next day went up to 300 mg/dl, with glucosuria and the extensive glucose metabolism evaluation confirmed the diagnosis of neonatal diabetes – high blood sugar (400 mg/dl) with glucosuria but no ketonuria and undetectable levels of insulin (1.4–1.6 mUI/l) and C peptide (0.14–0.16 nmol/l).

The insulin infusion was started (insulin diluted in a 1:100 mixture with normal saline), initially intravenously, trying to attain euglycemia and to get a positive growth evolution with consequent development of subcutaneous tissue. The insulin infusion rates were regularly titrated according to preprandial blood sugar levels, type of milk (breast or enriched formula), and postprandial (60 min) glycemia.

His initial insulin needs at 2.2 U/kg/day were relatively higher than those described in the literature, but the baby started to gain weight, between 30 and 50 g daily, and developed more subcutaneous tissue, mostly on thighs. Neurologically, he became more active.

**Figure 1:**
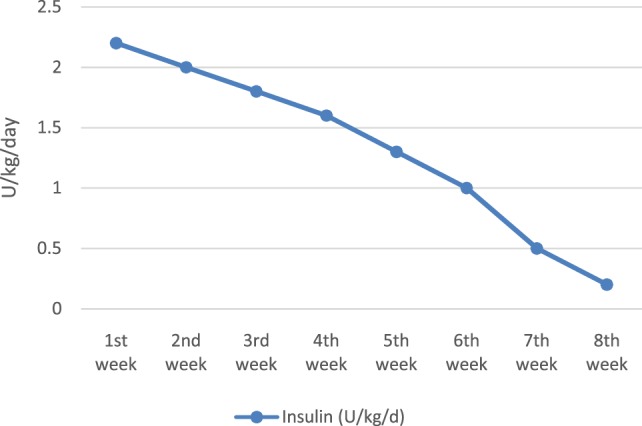
**Insulin requirement evolution**.

At the age of 17 days (11 days after diagnosis), when the subcutaneous tissue allowed us to adequately insert a catheter, the intravenous insulin infusion was switched to subcutaneous one (CSII), with no technical and skin problems in the evolution. The baby continued to improve his nutritional status (median daily weight gain of 30 g) and got more and more glycemic stability while reducing insulin requirements (Figure 1).

The insulin infusion was definitively stopped at the age of 8 weeks, when the amount was 0.2 U/kg/day and preprandial blood glucose levels between 60 and 70 mg/dl.

Almost 40% of total daily insulin dose was infused continuously as basal rate and the remaining 60% as mealtime boluses. Two different basal rates were set, one between the meals (0.05 U/h) and the second one during 1 h after each meal (0.07 U/h). Initially, the boluses were administered before each meal, but progressively, less frequent boluses were required (bolus if premeal blood sugar higher than 200 mg/dl). The amount of insulin infused as bolus was calculated using the insulin sensitivity (0.1 U to lower blood glucose by 40 mg/dl), and some adaptations were made depending on type of milk (human or enriched formula). At home, the basal rate was set at 0.06 U/h, and the boluses were infused if preprandial glycemia was above 150 mg/dl.

The blood glucose target was initially set between 100 and 250 mg/dl, trying to avoid the hypoglycemia-induced brain damage, particularly in newborn receiving exogenous insulin.

**Figure 2:**
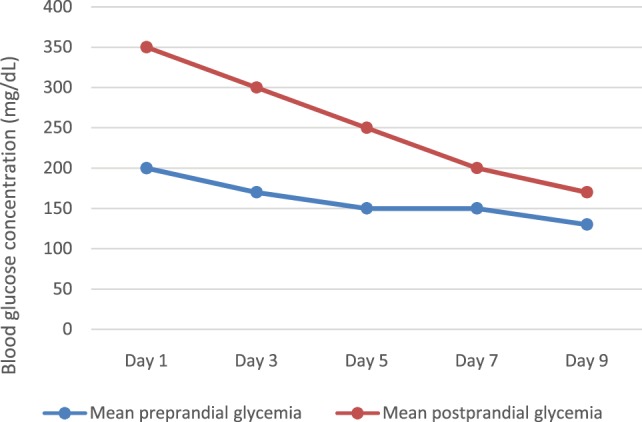
**Glycemic variations during intravenous insulin therapy**.

**Figure 3:**
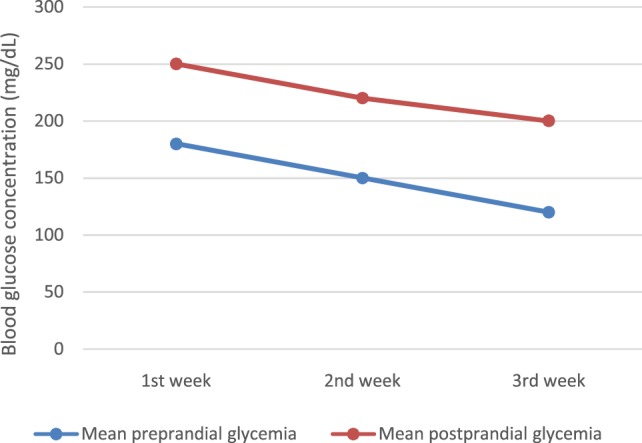
**Glycemic variations on CSII (the first 3 weeks)**.

Of course, we noted some glycemic excursions during the first days of intravenous insulin therapy (Figure 2), but after then, blood glucose variability decreased significantly, with almost all readings within the target range (100–250 mg/dl) (Figure 3). We changed progressively the target range, with a less wide target at the age of 4 weeks (at discharge) – 100–200 mg/dl.

There were no hypoglycemic accidents in his evolution. The use of continuous glucose monitoring (CGM) system was technically difficult; so the glycemic monitoring was mostly made by capillary sampling.

During the first 2 weeks, the baby was breastfed during the day, and during the night, he received 60 ml of enriched milk formula every 3 h (to prevent nocturnal hypoglycemia and assure a relatively higher-caloric intake recommended in newborn with IUGR). After 2 weeks, he was exclusively breastfed, with favorable weight gain.

The clinical suspicion of transient diabetes was more frequently evoked notably when, at the age of 4 weeks, the genetic results for permanent neonatal diabetes did not identify any mutation in INS, KCNJ11, and ABCC8 genes. From the fifth to eighth weeks of life, the infant presented a favorable catch up growth associated with a progressive decline of insulin infusion and stable metabolic profile. The clinical impression of evolution to a transient diabetes (decreasing needs of insulin with definitive stop at the age of 8 weeks) was soon confirmed by genetic testing performed at the Institute of Biomedical and Clinical Research, University of Exeter Medical School, showing a complete loss of methylation on chromosome 6q24 (paternal uniparental isodisomy).

A regular clinical follow-up was set up. At the age of 9 months, the baby develops very well, with normal anthropometric parameters, no infectious episodes and no hyper- or hypoglycemic events. The parents were informed about the necessity of long-term follow-up because the risk of recurrence is not insignificant and there are no clinical, biological, or genetic correlations to predict a possible recurrence.

The most important baby’s characteristics are briefly depicted in Table 1.

**Table 1:** **Newborn data summary**.

Birth parameters	GA (weeks)	Weight (g/percentiles)	Height (cm/percentiles)
	37	1980 (<3rd P)	43 cm (<3rd P)
Diagnosis	Age	Weight	Biological parameters
	6 days	1890 g	Glycemia 400 mg/dl
			Glucosuria++
			No ketonuria
			Insulin 1.4–1.6 mUI/l (2–17)
			C peptide 0.14–0.16 nmol/l (0.37–1.47)
Insulin therapy	Intravenous	CSII	Age of discontinuation
	From 6 to 17 days of life (total of 11 days)	From 17 days to 8 weeks of life	8 weeks
Genetic diagnosis	PND excluded (no INS, KCNJ11 and ABCC8 gene pathogenic mutations)	TND confirmed (complete loss of methylation on chromosome 6q24 – paternal uniparental isodisomy)	
	Age of 6 weeks	Age of 8 weeks	
Clinical follow-up (months)	3	6	9
	Weight 5400 g (15th P)	Weight 6820 g (3rd–15th P)	Weight 8000 g (15th P)
	Length 58 cm (15th P)	Length 64 cm (15th P)	Length 69 cm (15th P)
	Head circumference 36 cm	Head circumference 42 cm	Head circumference 44.5 cm (15th P)

*P, percentile; CSII, continuous subcutaneous insulin infusion; PND, permanent neonatal diabetes; TND, transient neonatal diabetes; INS, insulin*.

## Discussion

Clinical and molecular insight into neonatal diabetes has greatly advanced, and nowadays, we describe it as a monogenic disease and relationship between genotype and phenotype emerges.

On the clinical ground, it is very difficult to clearly differentiate the two types of neonatal diabetes. There is also a great genetic heterogeneity associated with neonatal diabetes, and important advances are currently made in defining some phenotype–genotype correlations.

For the transient neonatal diabetes, as genetic form of pancreatic β-cell dysfunction, two distinctive genotype–phenotype associations are defined. The 70% of cases are secondary to methylation abnormalities at chromosome 6q24 (paternal uniparental disomy, paternal duplication, loss of methylation without a structural defect), present with moderate-to-severe intrauterine growth retardation and develop early (during the first week of life) isolated moderate non-ketotic hyperglycemia. Despite this initial severe clinical appearance, the remission is evident before the age of 3 months and later relapse is possible. Few cases of transient diabetes are associated with functional abnormalities of K_ATP_ channels on the β-cell membrane (activating mutations of KCNJ11 and ABCC8 genes), and some clinical particularities could be retained, as higher weight at birth, later onset of hyperglycemia, and relatively retarded remission (2).

New genetic subtypes, including some pancreatic development transcriptions factors (GATA6, PAX6, NEUROG3, and NEUROD1), were recently described, particularly those linked to a phenotypic spectrum comprising diabetes and extrapancreatic features (3).

Today, even if the molecular diagnosis in babies with neonatal diabetes is quickly available and allows a better therapeutic and prognosis delineation, the initial clinical presentation (with all clinical and biological details) remains helpful for initial gene-candidate selection through molecular analysis.

In our case, we had all the clinical criteria to argue for transient diabetes: weight at birth, age when first hyperglycemia was detected, mild macroglossia and no other particular features, normal neurological status, relatively high starting doses of insulin with progressive diminution, and definitive stop before the age of 3 months. Additionally, at the age of 4 weeks, the first genetic result excluded the most frequent mutations associated with permanent diabetes. The genetic confirmatory results were available at the age of 8 weeks, and at this age, we have already stopped the insulin therapy.

While waiting for the genetic results, the medical treatment is the same and must target first an appropriate caloric intake to improve the nutritional status of these babies and insulin administration for an adequate blood sugar control (4, 5). Insulin is absolutely required and should be started as soon as the diagnosis was made. The way of administration and the amount required can vary depending on some clinical and technical aspects. All these babies had suffered a certain growth restriction, so their subcutaneous tissue is not adequately developed at birth and insulin absorption can be excessively erratic. In such conditions, intravenous insulin infusion could be the most practical option for initiating the insulin therapy. We started by intravenous access, with the continuous infusion rate and the preprandial boluses calculated depending on the premeal blood sugar level, milk formula type, and postprandial glycemia, trying to minimize the risk of hypoglycemia and glycemic variations. Eleven days later, after obtaining a stable glucose level and having more subcutaneous tissue available, we installed the pump and the baby returned at home at the age of 4 weeks. We appreciate the CSII could be an accurate, almost physiologic method to infuse insulin in newborns, and its major benefit was the ability to follow, flexibly, the baby’s blood glucose fluctuations.

In conclusion, neonatal diabetes is still a challenging entity, clinically, therapeutically, and genetically. From a clinical point of view, early diagnosis and medical management (continuous insulin infusion and high caloric intake) are required to avoid serious metabolic complications and allow adequate weight gain and neurological development in these moderate-to-severe growth-restricted babies. Clinical, biological, and evolution characteristics are useful tools in defining the molecular diagnosis. For babies with transient diabetes, regular follow-up, particularly in the first years of life is highly recommended because symptomatic hypoglycemia or recurrent hyperglycemia can be present during banal infections events (6).

## Ethics Statement

This case report was approved by the Ethics Committee of the Centre Hospitalier Regional de la Citadelle. The parents of the child gave written informed consent in accordance with the Declaration of Helsinki.

## Author Contributions

Study conception and design – CN. Acquisition of data – KF and JF. Analysis and interpretation of data – CN, JF, and VH. Drafting of manuscript – CN and VH. Critical revision – VH, JF, and CN.

## Conflict of Interest Statement

The authors declare that the research was conducted in the absence of any commercial or financial relationships that could be construed as a potential conflict of interest.
